# Incessant ovulation: a review of its importance in predicting cancer risk

**DOI:** 10.3389/fonc.2023.1240309

**Published:** 2023-10-06

**Authors:** Daniel W. Cramer

**Affiliations:** Department of Obstetrics, Gynecology and Reproductive Biology, Brigham and Women’s Hospital, Harvard Medical School, Boston, MA, United States

**Keywords:** ovulatory years, gynecologic cancer, breast cancer, testosterone, estradiol

## Abstract

Estrous cycles are recurring changes in therian mammals induced by estrogen, progesterone, and other hormones culminating in endometrial proliferation, ovulation, and implantation if fertilization occurred. In women, the estrous cycle is the menstrual cycle; but, unlike most mammals, the end of an infertile cycle is marked by endometrial sloughing and the start of another without an anestrous phase. Women stop cycling at menopause, while in most mammals, cycles continue until death. Epidemiologic studies identified menarche, menopause, births, lactation, and oral contraceptive (OC) use as key risk factors for ovarian, breast, and endometrial cancers. A composite variable was created to estimate the number of cycles not interrupted by events that stop ovulation. Captured by the phrase “incessant ovulation”, repetitive cycles were first postulated to affect ovarian cancer risk and later extended to breast and endometrial cancers. These associations could be explained by cumulative effects of repetitive tissue changes within reproductive organs, immune consequences of repetitive ovulation through the glycoprotein mucin 1, and residual effects of past ovulations that enhance ovarian production of testosterone. The latter two pathways could affect the risk for cancers in other organs not considered “reproductive”.

## Introduction

Estrous cycles are recurring events in placenta-forming mammals induced by estrogen, progesterone, and other hormones that culminate in changes to the uterine lining, ovulation, and support for an implanted zygote if fertilization occurred. Nutritional, environmental, and genetic factors determine the onset of sexual maturity, which occurs relatively later in animals with greater longevity and requires a certain body size to be achieved on an allometric scale. Estrous cycles vary in duration, frequency, and seasonality and, in most mammals, repeat until death (1). In women, the estrous cycle is the menstrual cycle; but unlike in most mammals, the end of an infertile cycle is marked by the sloughing of the endometrial lining and the immediate start of another without an anestrous phase (2). Women are polyestrous with an average cycle length of 28 days repeating until menopause. Ages at the first and last periods are important landmarks for the beginning of sexual maturity and the end of reproductive potential. With average ages at menopause and menarche approximately 51 and 12, the reproductive span is approximately 39 years.

The variable, ovulatory (or menstrual) years, is estimated by subtracting the age at menarche from the age at menopause (or current age if the woman is premenopausal) from which time pregnant, nursing, or using oral contraceptives (or other hormones stopping ovulation) are subtracted. Lifetime ovulatory (or menstrual) cycles are estimated by multiplying ovulatory years by cycles per year, approximately 13, for a cycle length of 28 days. More ovulatory cycles and greater cancer risk were first linked to ovarian cancer and later extended to breast and endometrial cancers. Here, we review the strength of the epidemiologic evidence for ovulatory years as a risk factor for these cancers, discuss mechanisms by which they might increase risk, and briefly examine how these mechanisms could link ovulatory cycles to cancers in organs not considered “reproductive”.

## Reproductive events and risks for cancers of reproductive organs

Globally, in 2020, it was estimated that breast, endometrial, and ovarian cancers totaled approximately 3 million new cases and a million deaths (3). Worldwide, incidence and mortality rates for breast, endometrial, and ovarian cancers are highly correlated (4); there is a greater-than-expected number of endometrial and ovarian cancers that occur after a diagnosis of breast cancer and a greater-than-expected number of breast cancer(s) after a diagnosis of ovarian or endometrial cancer (5). This suggests, and is the case, that ovarian, breast, and endometrial cancers share many common risk factors, especially related to reproductive events (**Table 1**).

**Table 1:** Reproductive events and risk for ovarian, breast, and endometrial cancers.

Hormones and contraception				
Factor	Ovary	Breast	Endometrium	Ref
Menarche	Late age ↓ risk	Late age ↓ risk	Late age ↓ risk	(6–11)
Cycle characteristics	↓ Risk with long and irregular cycles	No clear association	↑ risk with irregular cycles	(12–16)
Oral Contraceptives	↓ Risk	↑ Risk in younger women	↓ risk	(17–23)
Parity	↓ Risk	↓ Risk	↓ risk	(24–34)
Age at first/last birth	Early first and late last ↓ risk	Early first ↓ risk	Late first and late last ↓ risk	(24, 26, 29–36)
Breastfeeding	↓ Risk	↓ risk	↓ Risk	(37–41)
Menopause	Late ↑ risk	Late ↑ risk	Late ↑ risk	(8, 42–44)

"↑" indicates increased risk and "↓" indicates decreased risk.

Later age at menarche reduces the risk for all three cancers (6–11), but no single pattern of cycle regularity or length appears to affect risks in the same way. For ovarian cancer, long (>35 days) or irregular cycles reduce the risk for most histologic types (12). However, the disorder polycystic ovarian syndrome (PCOS), characterized by irregular, anovulatory cycles, does not affect the risk for ovarian cancer overall (13), while PCOS markedly increases endometrial cancer risk (14). The evidence is inconsistent about the effect of irregular cycles and PCOS on breast cancer risk (14–16). There is unequivocal evidence that the use of oral contraceptives (OCs) reduces the risk for ovarian and endometrial cancers (17–20). Some studies (21, 22), but not all (23), suggest that OCs may increase the risk for breast cancer in premenopausal women. For all three cancers, childbirth lowers risk (24–33); an earlier age at first birth reduces the risk for both ovarian and breast cancers (24, 26, 29–34). However, for ovarian cancer, there is evidence that a later age at and shorter interval since the last birth may lower risk more than early age at first birth (35), which also appears to be the case for endometrial cancer (36). Breastfeeding decreases the risk for all three cancers proportional to the length of breastfeeding (37–41). Finally, late age at menopause increases the risk for all three cancers (8, 42–44).

Not included in **Table 1** are reproductive events that do not or would not obviously stop ovulation such as tubal ligation, use of intrauterine devices (IUDs), and menopausal hormone use. Tubal ligation is clearly associated with reduced risks for ovarian cancer (45) and, possibly, endometrial cancer (46), but not breast cancer (47). IUD use, even if inert (not progestogen-secreting), clearly reduces the risk for endometrial cancer (48), but limited data suggest no clear effects on breast and ovarian cancers including the progestogen-secreting types (49–51). The literature on menopausal hormones and risks for breast, endometrial, and ovarian cancers is extensive. At the risk of over-simplification, estrogen-only preparation increase the risk for endometrial cancer and certain types of ovarian cancer, while breast cancer risk principally involves combination therapy. We provide a brief comment on the types of oral and non-oral hormonal contraception in relation to ovarian cancer. More contemporary, lower-dose, combination OCs are associated with a decrease in ovarian risk but less so for progestogen-only types (52). Alternatively, based on the equivalent duration of use, Depo-Provera injections may confer a greater reduction in risk than combined OCs, possibly related to residual anovulation that may follow its cessation (53).

## Estimated ovulatory cycles and risk for reproductive cancers

While epidemiologic studies of reproductive cancers have mostly focused on the effects of individual reproductive events, interest in a composite variable that combines these events has emerged. Before discussing the evidence linking ovulatory years and cancer risk, we consider issues related to constructing the algorithm to estimate them. Yang et al. evaluated 14 algorithms in relation to ovarian and endometrial cancers (54). They identified five similar algorithms and, among controls, found a high correlation of estimates. All five algorithms used ages at menarche and menopause (or current age) and number of births, while later models added durations of OC use and breastfeeding, number of miscarriages and abortions, and cycle length. Rules for linking anovulatory time with pregnancies varied with some using actual time pregnant or assigning times depending on the outcome—commonly 0.75 or 1 year for term pregnancies and 3 months for a miscarriage or abortion. Women who had a hysterectomy (without removal of ovaries) prior to menopause were either excluded or assigned an average age (approximately 50) for the last period. Women who “always” had irregular periods were either excluded or placed in a separate category.

Another issue relates to co-variates that should be adjusted for in estimating the risk for cancers that might be associated with the length of ovulatory years (LOY). We think it is unnecessary to adjust for the component variables used in calculating ovulatory years since these are already part of the variable. Adjusting for variables that are components of LOY weakens estimates of LOY’s effect on disease risk (and vice versa) (54). In addition to demographic factors, the two important variables that should be considered are body mass index (BMI) and smoking history. Women with greater BMI report earlier menarche (55); greater BMI increases the risk for certain histologic types of ovarian cancer (56), postmenopausal breast cancer (57), and endometrial cancer, markedly so (58). Current and former smoking is associated with earlier menopause (59), increased risk for mucinous ovarian cancer (60), no clear effect on breast cancer risk (61), and decreased risk for endometrial cancer (62).

Citing a study in which ovarian adenocarcinomas developed in hens housed to maximize egg production, Fathalla (63) first linked ovulation to ovarian cancer, coining the phrase “incessant ovulation”. First, to address the hypothesis, Casagrande et al. calculated ovulatory years from ages at menarche and last period minus “protected time” (i.e., births, incomplete pregnancies, and duration of OC use) (64). More ovulatory years increased the risk for ovarian cancer and its inverse, protected time, decreased risk. Additional studies followed confirming the association—some larger ones are noted here (54, 65–68). Associations are stronger with invasive serous, endometrioid, and clear cell cancers and weaker with low-grade or borderline serous tumors and mucinous cancers (68).

Since ovulatory years are not fully “set” until menopause, this might suggest that the association with ovarian cancer will be more apparent after menopause. In fact, the association is stronger in premenopausal women (67). This is also seen with its component variables where the protective effects of pregnancies, OC use, and breastfeeding are stronger in premenopausal than postmenopausal women (67). Purdie et al. reported that more ovulatory years accumulated when women were 20–29 carried a greater risk for ovarian cancer than the number of ovulatory years (in equivalent categories) accumulated at ages 30–39 or 40–49 (66). This suggests that more ovulations earlier in reproductive life carry a greater risk for ovarian cancer occurring before menopause. If so, the reproductive experiences of these women will have been subtracted from those of women who develop ovarian cancer after menopause, accounting for their apparently lower risk with ovulatory years. This would be an example of the depletion of susceptible individuals.

There are numerous data that breast cancer risk is increased by an earlier age at menarche and a later age at menopause (8), and subtracting the two also predicts risk. Longer total menstrual years (69) or longer menstrual years prior to a first livebirth (70) was linked to increased breast cancer risk. A 1984 paper argued that “sterile” menstrual cycles should be distinguished from total menstrual cycles by subtracting time pregnant, lactating, or using OCs to better estimate breast cancer risk (71). Two cohort studies provide evidence that more “sterile” menstrual cycles do increase breast cancer risk. One study found that either total cycles or total cycles prior to a first livebirth significantly increased breast cancer risk (72). Notably, this study examined the effect of lifetime menstrual cycles in models that either included or excluded OC users. The former model was slightly more significant than the model excluding OC users, suggesting that counting OC use adds to the protective effect of other factors that prevent ovulation. A second study confirmed increased breast cancer risk with lifetime (sterile) menstrual cycles but not with the number of cycles prior to a first livebirth (73). Both studies adjusted for BMI but not smoking. The effect of lifetime cycles on types of breast cancer has not been studied, although the large meta-analysis of the effects of earlier menarche and later menopause was most apparent for lobular and estrogen receptor-positive breast cancer (8).

Although PCOS clearly increases endometrial cancer risk (14), there is good evidence that repeated (but regular) ovulatory cycles also increase risk. A case–control study by Pettersson et al. found that a menstrual span of more than 40 years (subtracting births and OC use) led to a fourfold increase in risk compared to women with <25 years (74). Additional case–control and cohort studies found that a longer menstrual span, subtracting at least pregnancies, was associated with a significantly elevated risk for endometrial cancer (54, 75, 76). Notably, the last study showed the association pertained to women with high and low BMI and, also, adjusted for smoking (76). Although this study tabulated data to illustrate that a shorter menstrual span is protective relative to a longer one, their data translate to a nearly identical finding as Pettersson’s study (74); i.e., 40 or more menstrual years equates with about a fivefold increase in risk relative to women with <25 menstrual years.

Thus, LOY is a risk factor common to ovarian, breast, and endometrial cancers. Mechanisms that could explain these associations could either be organ-specific or based on common pathways.

## Ovulatory cycles: tissue effects

A menstrual cycle is divided into a follicular phase, where follicle-stimulating hormone (FSH) promotes a cohort of developing follicles to secrete estrogen and the endometrium proliferates; ovulation, where the dominant follicle releases its oocyte associated with a spike in FSH and luteinizing hormone (LH); a luteal phase, where the granulosa-thecal layer from the dominant follicle is converted to a progesterone-secreting corpus luteum and the endometrium is converted to a secretory type; and menstrual phase, where the endometrium is shed and a new cohort of follicles selected, if fertilization did not occur (77). The superficial two-thirds of the endometrium (decidua functionalis) is the zone that proliferates, undergoes secretory transition, and sheds if no implantation occurred. The deepest region (decidua basalis) does not undergo significant proliferation but is the source of endometrial regeneration after each menses (78). Tissues undergoing large numbers of cell divisions have a greater likelihood of DNA replication errors compared to tissues with less-active divisions. Estimates of the rate of sporadic mutations in human cells, on the order of 10^−7^ mutations per gene per cell division (79), suggest that the number of cells with “first hits” on a carcinogenic pathway may number in the hundreds for every gram (approximately 10^9^ cells/g) of proliferating tissue (80). Sporadic mutations of the PTEN tumor suppressor gene, associated with type 1 endometrial cancer, are observed in almost half of histologically normal endometria of naturally cycling women (81). While most OC users do have periods, the endometrium is exposed to progestins for a longer duration, which may decrease the likelihood of survival of PTEN mutant clones in the endometrium (82, 83).

The breast also undergoes cyclic changes, especially involving the terminal ductal lobular units thought to be the source of both lobular and ductal breast cancer (84). Similar to using morphologic changes to date the endometrium during a cycle, changes in breast lobules were considered to correlate with the cycle phase. A study of surgical specimens from premenopausal women with benign breast disease not currently using OCs analyzed the degree of epithelial–myoepithelial distinction, vacuolation, stromal edema, apoptosis, and mitosis as key features (85). Scores were the highest in the late luteal phase, indicating a greater degree of proliferation. Immunohistochemical studies using the Mib-1 antibody to detect the proliferative marker, Ki67+, confirmed this (86). Ki67+ cells were also counted in normal breast tissue in Nurses’ Health Study (NHS) participants with prior biopsies (for benign disease) and demonstrated that women with higher counts had an increased risk for future breast cancer (87). These data suggested to Brisken the direct effects of progesterone (in combination with high estradiol) on breast epithelium (88). However, progesterone and estradiol could be acting indirectly through the action of other hormones elevated in the luteal phase including growth hormone (89). In any case, exposure to a greater number of luteal phases could explain why more cycles increase breast cancer risk.

Proliferative changes in the ovary during a cycle primarily involve stromal, granulosa, and thecal cells that surround immature oocytes and promote them to develop into mature oocytes. However, cancers arising from granulosa, thecal, or germ cells are relatively rare in adult women in whom the predominant types are epithelial tumors. The surface epithelium of the ovary was thought to be the origin of these tumors, and repeated disruption and repair of this layer with each ovulation might lead to p53 mutations, predisposing to epithelial ovarian cancer where defective p53 is a hallmark feature (65). However, a subsequent study did not confirm that those ovarian cancers most clearly linked to a greater number of ovulatory cycles were more likely to be p53 positive (90). Nevertheless, surface epithelial disruption and implantation of endometrial cells shed through the fallopian tubes during menses might be involved in the pathogenesis of other types of ovarian cancer such as clear cell and endometrioid (91).

Around the turn of the century, secretory cells in the distal (fimbriated) end of the fallopian tubes (FTSEC) began to receive attention as the source of high-grade serous ovarian cancers, the most common and lethal type (92). A case was made for the progression of FTSEC with accumulating defective P53 advancing to lesions called serous tubal intraepithelial carcinoma (STIC) (93). STIC could rapidly progress to stage III ovarian cancer by shedding malignant cells into the pelvic cavity to implant. However, rather than the cyclic proliferation of FTSEC causing precursor lesions, repeated exposure of FTSEC to follicular fluid (FF) with ovulation might be the instigating factor. FF contains growth factors, cytokines, and reactive oxygen species (ROS) capable of inducing DNA damage (94). *In vitro* studies demonstrated that exposure of tubal cells in culture to FF led to the proliferation of FTSEC and P53 accumulation (95), suggesting inflammation as the basis for this progression. Inflammation as a common pathway for cancer, in general, was proposed by Cousens and Werb (96) and for ovarian cancer, in particular, by Sanchez-Pietro et al. (97). This paper was entitled “Etiopathogenesis of ovarian cancer. An inflamm-aging entity?” and reviews the broader role of inflammation in ovarian cancer. However, we point out that the association between ovulation and ovarian cancer is not a simple reflection of more ovulations equals greater age, since age was adjusted for in examining this association.

In addition to the cumulative effects of ovulatory cycles on tissues specific to the breast, endometrium, and ovaries, there are mechanisms that offer more general explanations for LOY as a risk factor for reproductive cancers and, perhaps, other cancers. These include an immune-based model and a hormonal model.

## Ovulatory cycles: immune effects involving MUC1

An immune-based model involves human mucin 1 (MUC1), a transmembrane-glycoprotein expressed at the apical cell surfaces of normal glandular epithelium of many organs including breast, salivary glands, gastrointestinal tract, pancreas, bladder, prostate, fallopian tubes, endometrium, and some hematopoietic cells (98). In cancers arising from ductal epithelium, MUC1 becomes overexpressed on the entire cell surface, and a less-glycosylated form of MUC1 is produced. This overexpression favors metastases through several mechanisms: dampening immune response; interacting with other transmembrane proteins, like EGFR; and engaging cytoplasmic-signaling proteins such as Src and beta-catenin (99). Elevated levels of MUC1 are associated with poor prognosis for breast, prostate, ovarian, gastric, bile duct, colon, and non-small cell lung cancers (99). MUC1 is detected by assays based on antibodies against various MUC1 epitopes leading to a variety of names in the literature including CA15.3, DF3 antigen, human milk fat globule (HMFG) antigen, epithelial membrane antigen (EMA), polymorphic epithelium mucin (PEM), and episalin (98).

Assays to measure anti-MUC1 antibodies have also been developed using various MUC1 epitopes associated with tumor-like MUC1 (100). IgM and IgG anti-MUC1 antibodies have been identified in patients with various adenocarcinomas, and their presence has been associated with better prognosis in breast cancer (101), colorectal cancer (102), non-small cell lung cancer (103), and pancreatic cancer (104). These observations prompted interest in immune-based treatments involving MUC1 including vaccines based on MUC1 epitopes coupled with various adjuvants, passive immunization with anti-MUC1 antibodies, transfer of MUC1-specific cytotoxic T cells, and others (105). Most trials involve individuals with advanced cancer, but at least one trial involved patients with colon adenomas at risk for colorectal cancer, demonstrating that high levels of anti-MUC1 IgG could be achieved with a MUC1-based vaccine except in individuals with evidence of pre-existing circulating myeloid-derived suppressor cells (106).

Natural antibodies against MUC1 have also been found in healthy individuals, raising important questions. What events are associated with the appearance of anti-MUC1 antibodies, and do these antibodies contribute to successful immunosurveillance of MUC1+ cancers? Anti-MUC1 antibodies generated in the absence of cancer appear to be promoted by inflammatory events involving tissues that normally produce MUC1. These events may include childhood mumps, puerperal mastitis, and tubal ligation—each shown to raise anti-MUC1 antibodies and each shown to lower the risk for ovarian cancer in case–control studies (107–109). Importantly, there are prospective data from two studies suggesting that natural antibodies against MUC1 can lower the risk for ovarian cancer. Data from the NHS showed that women with higher anti-MUC1 antibodies subsequently had a lower risk for ovarian cancer developing before age 65 (110), and data from the EPIC cohort showed that elevated levels of anti-MUC1 antibodies may lower the risk for serous ovarian cancers arising within 3 years of the blood draw (111). More data on antibody levels at various times after the event that provokes antibodies are necessary to support this premise.

Pointing out that the presence of anti-MUC1 antibodies leads to reduced risk for ovarian cancer, the corollary follows that their absence may increase the risk for cancer. We have shown that a greater number of ovulatory cycles are associated with lower anti-MUC1 antibody levels in three different populations using two different assays to measure anti-MUC1 antibodies (68, 110, 111). We previously interpreted this as indicating that repeated exposure to MUC1 from the endometrium during the luteal phase of cycles may lead to a downregulation of MUC1 immunity and lower antibody levels (68), but it is also possible that lower anti-MUC1 antibody levels with repeated cycles result simply from absence of those events known to raise antibody levels such as pregnancies and breastfeeding. In either case, this brings us to the relevance of MUC1 immunity to the topic of this paper—incessant ovulation. As a common explanation, immunity related to human mucin 1 could explain the risk for not only ovarian, breast, and endometrial cancers but also other MUC1-expressing cancers that have been targets of MUC1 vaccine trials (104), such as colorectal cancer and lung cancer, and point to the possible relevance of ovulatory cycles to their epidemiology.

## Ovulatory cycles: hormonal effects

Prospective studies found that the combination of elevated serum testosterone and estradiol in postmenopausal women predicts future risk for endometrial, breast, and ovarian cancers (112–115). Relating this pattern to the number of past ovulations requires answers to the following questions. What is known about the hormone-producing potential of the postmenopausal ovary? What biological events occurring within the ovary during an ovulatory cycle might enhance later hormone production? Finally, is there any direct evidence that LOY does, in fact, affect hormone levels in postmenopausal women?

The answer to the first question comes from studies in which ovarian veins were cannulated in postmenopausal women at the time of hysterectomy and/or oophorectomy and levels of sex hormones coming directly from the ovaries compared with levels in the peripheral blood (116–118). These studies demonstrated substantially higher levels of testosterone in the blood from the ovaries compared to peripheral levels with gradients up to 20-fold higher and persisting in women even 20 years past menopause. Cannulation studies were also performed in women with ovarian and endometrial cancers and women with breast cancer later undergoing oophorectomy and showed even higher gradients of hormone levels, especially testosterone, compared to women without cancer (119–121). Because levels of testosterone and androstenedione dropped postoperatively (118), the cannulation studies counter the argument that the adrenals are the primary source of androgens in postmenopausal women (122). Cross-sectional studies show that women who had bilateral oophorectomy prior to menopause have low testosterone levels when compared to those with intact ovaries (123).

Biological events during an ovulatory cycle that might change the ovary’s potential for androgen production likely involve the ovary’s germ cell reserve, the process of follicle maturation, and the fate of follicles recruited during a cycle but not selected for ovulation (124, 125). Primordial follicles are oocytes surrounded by a single layer of pre-granulosa cells. The maximum quota of primordial follicles, likely more than a million, is established at birth of which approximately 300,000 remain when puberty begins. During each menstrual cycle, a cohort of follicles is selected to undergo maturation involving the growth of the granulosa layer, formation of a thecal-cell layer, secretion of follicular fluid to form antral follicles, and selection of, usually, just one to become a Graafian follicle from which the oocyte will be released. Remnants of the dominant follicle then form the corpus luteum, which produces progesterone to maintain early pregnancy but will regress without a fertilized oocyte. During a cycle, some follicles that reach the early-antral stage will become quiescent and remain at that stage as a more dynamic reserve than primordial follicles. However, the remainder of those follicles that developed further during a cycle will undergo apoptosis to become atretic follicles. Follicular atresia is likely most relevant to the consequences of repetitive ovulation (126). Evidence that an antral follicle is on the path to atresia is apoptosis within the granulosa cell layer. Estradiol drops from both a decrease in granulosa cell number and reduced aromatization of androgens secreted by thecal cells into estradiol. Thecal cells remain viable after the disappearance of granulosa cells and retain their ability to secrete androgens, but their longer-term fate is unclear.

Possibly relevant to the fate of thecal remnants are two related conditions called ovarian stromal hyperplasia (OSH) and hyperthecosis (OHT). OSH refers to the nodular or diffuse proliferation of the stromal cells of the ovary, while OHT is distinguished by the presence of round to polyhedral theca-lutein-like cells occurring singly or within nests within a stroma that is itself proliferative (127). For decades, these conditions have been of interest in the context of describing ovaries removed from women with endometrial cancer (128). A link between OSH and breast cancer has also been reported (129); case reports describe OHT at the edge of epithelial ovarian malignancies (130). A study that linked elevated testosterone to OSH involved (premenopausal) women presenting with hirsutism who had oophorectomy or wedge resection. Cannulation studies like those described earlier were performed and revealed elevated testosterone and dihydrotestosterone levels in the blood from ovaries, especially from ovaries with more extensive OHT and larger gradients compared to peripheral levels compared to women without OHT (131).

Given this background, is there evidence that LOY influences postmenopausal hormone levels? Two studies in the current literature address this with conflicting results (132, 133). The most recent paper used data from the NHS on hormone levels measured in the blood collected from 1,976 healthy postmenopausal women in 1990 (133). This study excluded women who had bilateral oophorectomy but included hysterectomized women who retained at least one ovary. Age at menopause was imputed in the latter group. In this study, greater LOY was associated with higher testosterone as well as greater estradiol in those women with above-average BMI. This could reflect the accumulation of functioning stromal and thecal cells from repeated ovulation that secrete testosterone, which is converted to estradiol peripherally. An earlier study of 860 postmenopausal women also looked at hormonal levels in relation to lifetime ovulatory cycles (132). This study did not find a relationship between the number of cycles and testosterone or estradiol levels. Adjustment variables, similar to NHS, were used in this study except that OC use was adjusted for, although it was already part of the algorithm to calculate cycles. As mentioned earlier, adjusting for variables that are already components of LOY weakens estimates of its effect on disease risk. In addition, compared to NHS, this study was smaller and had a higher percentage of nulliparous women with lower parity, greater use of birth control pills, and more current smokers. Finally, there was a greater prevalence of hysterectomized women who were excluded in this study. There are many cross-sectional studies of hormone levels in postmenopausal women with sufficient data on reproductive variables to calculate LOY and confirm whether, in fact, more LOY leads to higher testosterone.

We pointed out that the model involving a link between LOY and mucin immunity may pertain to cancers in “non-reproductive” organs. The same may apply to the model involving LOY and sex hormones. Melanoma may be such a cancer. A large prospective study of melanoma in French women looked at ovulatory age and found that postmenopausal women with LOI <33 years had half the risk for melanoma compared to women with LOI >39 years, RR (and 95% CI = 0.51 (0.28, 0.91), equating with greater risk for more ovulatory years (134). Details for constructing the algorithm were not discussed but, apparently, did not include OC use. An earlier case–control study also looked at ovulatory years using a more detailed algorithm that included ages at menarche and menopause (or current age) reduced by 1 year for each pregnancy and total years of OC use. This study also found a greater risk for melanoma associated with longer ovulatory years when adjusted for subjects’ estimated sunlight exposure (135), suggesting additive effects involving sun exposure and LOY. Given our contention that the key effect of past ovulations is on testosterone, this adds relevance to an experimental study reporting that S91 murine melanoma cells grow in response to testosterone and that this effect is modulated by light (136). Additionally, although case reports carry little scientific weight, one that reported the onset of dysplastic nevi in a postmenopausal woman who began using a transdermal patch with testosterone and estradiol that stopped after the patch was discontinued appears relevant (137).

## Summary and discussion

A somewhat unique feature of estrous cycles in women compared to other mammals includes the sloughing of the endometrial lining at the end of a cycle in which an oocyte was not fertilized. This allows estimates of the onset of sexual maturity, end of reproductive potential, and, in between, the frequency of presumed ovulatory cycles that were not interrupted by pregnancies, breastfeeding, or OC use. There are substantial epidemiologic data that support the premise that more estimated ovulatory cycles (captured by the phrase “incessant ovulation”) increase risks for ovarian, breast, and endometrial cancers. One pathway through which these effects may occur includes cumulative effects of repetitive tissue changes within reproductive organs with each ovulatory cycle. In the terminal ductal lobular units of the breast, proliferative activity is increased during the luteal phase of the cycle, which could be a key factor in both lobular and ductal breast cancer. In the endometrium, repetitive shedding and regrowth of the endometrium may lead to the accumulation of mutations in the tumor suppressor, PTEN, a marker for endometrial cancer. In ovaries, the secretory epithelium of the tubal fimbria, with each ovulation, is exposed to follicular fluid whose inflammatory properties may induce the proliferation of cells with defective p53 leading to high-grade serous cancers. Although the vagina and cervix also undergo cyclic changes, cancer involving these organs appears to be dominated by infectious agents. However, it is possible that tissue changes in other organs could be affected by repetitive cycles; ovulatory years should be examined for any cancer in which there are male–female differences in occurrence.

A second pathway might involve immune events related to human mucin 1, a transmembrane glycoprotein, expressed at the apical cell surfaces of normal glandular epithelium of many organs including the breast, fallopian tubes, and endometrium. In cancers arising from these tissues, MUC1 becomes overexpressed on the entire cell surface favoring metastases through several mechanisms and associated with poor prognosis. Conversely, some patients with MUC1-positive cancers have anti-MUC1 antibodies; their presence is associated with better prognosis. Anti-MUC1 antibodies have also been identified in healthy individuals without cancer, and there is some evidence to suggest these antibodies may lower the risk of ovarian cancer. Repetitive ovulation enters the story based on the observation that more ovulatory cycles are associated with lower anti-MUC1 antibodies, which are associated with increased risk for ovarian cancer. It is not clear, however, whether lower anti-MUC1 antibodies seen with more ovulatory cycles reflect immune downregulation from the cyclic elevation of MUC1 released during ovulation or menstruation or simply reflect fewer events like pregnancy and breastfeeding known to produce anti-MUC1 antibodies. This pathway could apply to cancer in which immune-based treatments involving MUC1 have shown some success including colon and small cell lung cancers. The role of ovulatory years in the epidemiology of these cancers should be investigated; the possibility that immune reactions are related to other mucins such as MUC16 (CA125) should be considered.

A third pathway involves residual effects of past ovulations that enhance the postmenopausal ovary’s production of testosterone, which can then be converted to estradiol in peripheral tissue, especially in women with greater BMI. This effect may result from the accumulation of residual stromal and thecal cells recruited during ovulatory cycles that display greater resistance to apoptosis than granulosa cells. Two observations support the relevance of this pathway to explain why more ovulations increase the risk for breast, endometrial, and ovarian cancers. First is the observation that a pattern of elevated testosterone and estradiol in healthy postmenopausal women is associated with a greater risk for these cancers. Second, the ovaries of women with breast and endometrial cancers display patterns described as OSH and OHT. Like the possible pathway involving the immune effect related to mucins, this pathway could support a credible link between more ovulations and cancer in non-reproductive organs including melanoma in which testosterone may play a role.

In conclusion, the estimation of ovulatory cycles deserves much greater attention as a factor in the epidemiology of cancer in women.

## Author contributions

The author confirms being the sole contributor of this work and has approved it for publication.
